# TNER: a novel background error suppression method for mutation detection in circulating tumor DNA

**DOI:** 10.1186/s12859-018-2428-3

**Published:** 2018-10-20

**Authors:** Shibing Deng, Maruja Lira, Donghui Huang, Kai Wang, Crystal Valdez, Jennifer Kinong, Paul A. Rejto, Jadwiga Bienkowska, James Hardwick, Tao Xie

**Affiliations:** 1 Pfizer Early Clinical Development Biostatistics, Cambridge, UK; 2 Pfizer Oncology R & D, San Diego, CA USA

**Keywords:** ctDNA, Next-generation sequencing, Variant calling, Error suppression, Single-nucleotide variant

## Abstract

**Background:**

Ultra-deep next-generation sequencing of circulating tumor DNA (ctDNA) holds great promise as a tool for the early detection of cancer and for monitoring disease progression and therapeutic responses. However, the low abundance of ctDNA in the bloodstream coupled with technical errors introduced during library construction and sequencing complicates mutation detection.

**Results:**

To achieve high accuracy of variant calling via better distinguishing low-frequency ctDNA mutations from background errors, we introduce TNER (Tri-Nucleotide Error Reducer), a novel background error suppression method that provides a robust estimation of background noise to reduce sequencing errors. The results on both simulated data and real data from healthy subjects demonstrate that the proposed algorithm consistently outperforms a current, state-of-the-art, position-specific error polishing model, particularly when the sample size of healthy subjects is small.

**Conclusions:**

TNER significantly enhances the specificity of downstream ctDNA mutation detection without sacrificing sensitivity. The tool is publicly available at https://github.com/ctDNA/TNER.

**Electronic supplementary material:**

The online version of this article (10.1186/s12859-018-2428-3) contains supplementary material, which is available to authorized users.

## Background

Cancer is a genetic disease that is driven by changes to genes controlling cellular function [1]. Characterizing the disease at the molecular level is essential for early detection, personalized therapy based on tumor genomic profiles, monitoring tumor progression and response to treatment and the identification of resistant mechanisms [2]. For solid tumors, tumor tissue biopsies are typically necessary to obtain samples for genotyping or other molecular analyses. Biopsy procedures are usually invasive and introduce additional risk to the patient’s health. In many cases, tumor tissue biopsy is contraindicated medically, and the tissue samples are often insufficient or unsuitable for molecular profiling [3]. In addition, cancer is a heterogeneous disease that can include different subclones within the same primary tumor and between the primary tumor and metastatic lesions. This heterogeneity in tumors can lead to variations in tumor tissue sampling through biopsy [4].

Both cancer and normal cells shed DNA as a result of apoptosis and other biological processes and release DNA fragments into the blood stream to become cell-free DNA (cfDNA) [5–7]. The cfDNA derived from tumor cells is called circulating tumor DNA (ctDNA) and provides a real-time genomic snapshot of cancer cells due to the relatively short half-life of cfDNA (~ 1–2 h) [2, 8]. Thus, ctDNA is a form of “liquid biopsy” that provides a noninvasive alternative to tissue biopsy for cancer diagnosis and monitoring [9, 10]. Moreover, ctDNA from all tumor lesions is generally pooled in the circulatory system; therefore, it can reduce the sampling variation associated with tumor heterogeneity in comparison to that of a single tissue biopsy [11].

The fraction of ctDNA in the total cfDNA in plasma, however, can be extremely low in many cancer patients [2, 8]. Recently established techniques, such as droplet-digital PCR (ddPCR), enable the detection and quantification of low-abundance ctDNA but cover only a small number of known “hotspot” mutations [8, 12]. Advances in DNA sequencing technology have made it possible to identify ctDNA mutations with sensitivity comparable to that of ddPCR [13, 14] when the sequence coverage is sufficient (> 10,000x per base). One of the most significant challenges in detecting ctDNA mutations is suppressing technical errors introduced during library preparation, PCR amplification and sequencing itself [15]. While errors arising during PCR amplification can be removed effectively using molecular barcodes [15], other technical errors are more universal and need to be removed before mutation calling [3, 16]. Newman et al. [17] recently proposed a creative integrated digital error suppression (iDES) method that includes both a molecular barcoding system to reduce PCR errors and a background polishing model with an improved estimation of background mutation error rate (BMER) compared to the previous computational method used in CAPP-Seq [18]. Specifically, the BMER was mostly estimated using a model of Gaussian distribution on the mutation data from a collection of healthy subjects [17]. To our knowledge, there are very few background polishing methods designed for ctDNA detection, and iDES is the only publicly available state-of-the-art method. The polishing method used in iDES increased the percentage of error-free positions from ~ 90 to ~ 98% (based on a 300 kb panel, Fig. 2b in [17]). However, approximately 6,000 positions containing a substantial number of noisy bases could still be misclassified due to the relatively small sample size (*n* = 12) of healthy subjects and the nature of the data (small discrete counts), which made it difficult for the Gaussian model to robustly estimate the background.

To provide a more robust estimation of background noise and remove the sequencing artifacts more effectively for panel sequencing data, we developed a novel background polishing method called TNER (Tri-Nucleotide Error Reducer) with a Bayesian consideration to overcome the small sample size issue. TNER is based on tri-nucleotide context data and uses a binomial distribution for the mutation error count to estimate the background from healthy subjects. The tri-nucleotide context (TNC hereafter) consists of 96 distinct substitutions in the specific context of the tri-nucleotide, consisting of the 6 distinguishable single-nucleotide substitutions (C > A, C > G, C > T, T > A, T > C and T > G) and the 16 possible combinations of immediately preceding and following bases. TNC has been extensively studied in cancer genetics to construct mutation signatures as a response to carcinogens (an excellent summary is available at http://cancer.sanger.ac.uk/cosmic/signatures), to compare the mutational spectra of trunk and branch mutations, and to predict the clinical implications of called mutations [19–21]. Given that the pattern of low-frequency technical errors from next-generation sequencing (NGS) should be similar in normal control samples and patient samples, we argue that local sequence context could help better model noise for a small sample size of healthy subjects by leveraging information from other bases with a shared TNC. The TNER methodology proposed here, to the best of our knowledge, is novel in this area. As an effective error reducer, TNER can be easily integrated into an existing variant-calling pipeline before the variant caller to detect very low-frequency mutations in liquid biopsy samples. TNER is freely available at https://github.com/ctDNA/TNER.

## Methods

### NGS data for analysis

To demonstrate the performance of the error suppression model in detecting single-nucleotide variations, we analyzed targeted sequencing data of plasma cfDNA from healthy subjects using a panel of 87 cancer genes (http://cancerres.aacrjournals.org/content/77/13_Supplement/2749). The barcoded target-enriched DNA library (147 kb) was sequenced on an Illumina HiSeq 4000 platform, generating ultra-deep coverage with an average coverage per base of ~ 12,000x.

### Tri-nucleotide error reduction model

The detection of ctDNA is typically achieved through detecting signature mutations associated with tumors in cfDNA. Sequencing data from cfDNA contain many stereotypical errors or other background mutation errors that are not of tumor origin [22]. To call a mutation in ctDNA, the distribution of the BMER needs to be characterized at each nucleotide base position to reduce false positive error, for example, by modeling cfDNA data on the same NGS panel from healthy subjects [17]. The mutation rates from healthy subjects are assumed to be background mutation noise associated with both technical and biological sources. One challenge in characterizing the individual nucleotide BMER from healthy subjects is the relatively small cohort size. The iDES method used 12 healthy subjects [17]; we used a comparably sized set of 14 healthy subjects. These small sample sizes do not allow a reliable estimate of the background error distribution for individual nucleotides. The Bayesian method with prior information can help to overcome this limitation.

To better estimate the BMER distribution, we propose a background error model originating from a hierarchical Bayesian method that utilizes the distribution of mutation error rate in a TNC, which consists of the mutated nucleotide and the combinations of immediately preceding and following nucleotides. Mutation signatures characterized by TNC have been used frequently in cancer genetics [19, 21, 23]. There are 96 distinct TNCs, and we assume that they are independent. For a nucleotide in TNC group *i* (*i* = 1, …, 96) at base position *j* (*j* = 1,…J), the number of background error reads *X*_ij_ observed for a given coverage *N*_j_ is assumed to follow a binomial distribution

1 $$ {X}_{\mathrm{ij}}\sim \mathrm{Binom}\left({N}_{\mathrm{j}},{\pi}_{\mathrm{ij}}\right) $$

with a position-specific mutation error rate parameter π_ij_. J is the total number of bases in the panel (147 k). With a large *N* (typically > 1,000) and a small π (< 1%), X can also be modeled as a Poisson distribution

2 $$ {\mathrm{X}}_{\mathrm{ij}}\sim \mathrm{Pois}\left({\mathrm{N}}_{\mathrm{j}}\ast {\uppi}_{\mathrm{ij}}\right) $$

with rate parameter N_j_ ∗ π_ij_. We will focus on the binomial model here.

The BMER at position j can be estimated using the average mutation error rate of the j^th^ base position from the 14 healthy subjects, ${\widehat{\uppi}}_{\mathrm{ij}}$. This position-specific parameter will be poorly estimated because of the small sample size. To improve the estimate of π (for simplicity we drop the subscription for now), we propose a Bayesian framework and assume that π follows a beta distribution within a TNC

3 $$ \uppi \sim \mathrm{Beta}\left(\upalpha, \upbeta \right) $$

The use of the beta prior is primarily due to its conjugation to the binomial distribution and its goodness of fit to the data (see Discussion). For convenience, we reparameterize the beta distribution using its mean as a parameter.

4 $$ \uppi \sim \mathrm{Beta}\left(\upmu, \upnu \right),\mathrm{with}\ \upmu =\frac{\upalpha}{\upalpha +\upbeta}\ \mathrm{and}\ \upnu =\upalpha +\upbeta $$

The prior parameters of the beta distribution can be estimated based on the BMER distribution of nucleotides in a TNC using the method of moments [24]. The mean parameter μ can be estimated by the average mutation error rate ($\widehat{\upmu}$) of nucleotides in the TNC. The ν parameter can be estimated using $\widehat{\upmu}$ and the sample variance of BMER within the TNC. For a position with a mutation count of *x* out of *n* total reads, the posterior distribution of the BMER at this position will be a Beta(α + *x*, β + n − *x*) with a mean parameter.

5 $$ {\mu}^{\prime }=\frac{\alpha +x}{\alpha +\beta +n}= w\mu +\left(1-w\right)x/n $$

where *w* = (*a* + *b*)/(*a* + *b* + *n*).

Therefore, the posterior mean of the position-specific BMER for position *j* with TNC *i* can be estimated with a shrinkage estimator, that is, a weighted average of the TNC level mutation error rate (${\widehat{\upmu}}_{\mathrm{i}}\Big)$ and the position-specific rate ${\widehat{\uppi}}_{\mathrm{ij}}$

6 $$ {\overset{\sim }{\pi}}_{ij}={w}_{ij}{\widehat{\mu}}_i+\left(1-{w}_{ij}\right){\widehat{\uppi}}_{\mathrm{ij}} $$

The weight *w*_ij_ can be derived in closed form under a beta-binomial distribution and estimated using the method of moments [25]. We found that the analytic Bayesian weight worked well for the vast majority of the positions except for a small number (< 1%) of positions where the estimated position-specific error rate ${\widehat{\uppi}}_{\mathrm{ij}}$ is large. In those positions, the shrinkage towards a smaller ${\widehat{\upmu}}_{\mathrm{i}}$ tends to underestimate the true background mutation error. Therefore, we adopted a modified weight that balances the relative size of the TNC error rate and the position-specific error rate

7 $$ {w}_{ij}=\frac{{\widehat{\mu}}_i}{{\widehat{\mu}}_i+{\widehat{\pi}}_{ij}} $$

This weight function provides less shrinkage when the position-specific mutation error rate is high - a property that helps retain the position-specific background when it is much higher than the tri-nucleotide level background. Although this simple weight does not reflect the impact of sample size, a larger sample size helps provide a better estimate of π_ij_. Due to this modification in weight, TNER adopted a more heuristic approach than a full Bayesian method.

Once we have an estimate of the BMER π_ij_ using Eq. (6), the threshold for mutation detection can be defined based on the upper posterior credible interval bound of π_ij_. At the α level, the upper 1-α/2 Clopper-Pearson interval bound for a binomial proportion is

8 $$ {B}_{ij}=\beta \left(1-\frac{\alpha }{2},{N}_j{\overset{\sim }{\pi}}_{ij}+1,{N}_j\left(1-{\overset{\sim }{\pi}}_{ij}\right)\right) $$

where β() is the quantile function of beta distribution; ${\overset{\sim }{\pi}}_{ij}$ is the posterior estimate of the mutation error rate in Eq. (6); and N_j_ is the average total reads for this position from healthy subjects. If the observed mutation error rate at position *j* with TNC *i* is lower than *B*_*ij*_, those variants mapped to the TNC will be classified as background noise and polished using the reference allele; otherwise, the variants will not be polished (possibly true mutations). In the Bayesian model, multiple comparison is not a major concern because the prior distribution allows pooling information between positions and avoids false positive calls when variation is low [26]. In our analysis, false positive calls are very rare when the method is applied to healthy subjects (see Results). A similar beta-binomial model has been used in other studies [27–29]. However, none of them used the model to estimate the BMER distribution with TNC, nor did they apply the model to ctDNA NGS data.

## Results

### Model performance on the healthy subject data

**Fig. 1:**
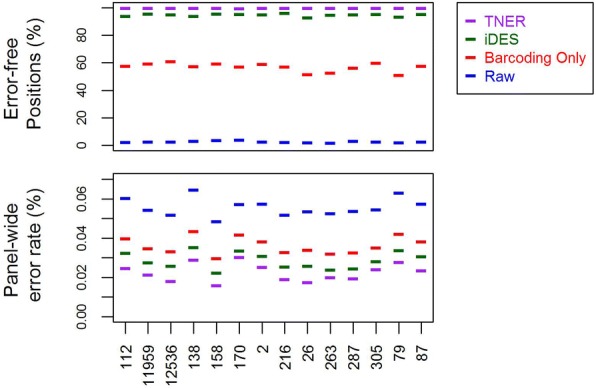
Error-free positions (%) and panel-wide error rate of the 14 healthy subjects’ data (sample labels on x-axis) from the leave-one-out analysis with different methods. “Raw” = raw data, “Barcoding Only” = Barcoding error reduction only

We first evaluated the TNER model on the healthy subject data using the leave-one-out method and compared its performance to that of iDES with the default settings [17]. We built the background model using data from 13 healthy subjects and predicted the mutation in the left-out subject. Similar to Newman et al. [17], we counted the number of error-free positions, defined as those positions with exclusively reference allele reads after error suppression, for each of the 14 healthy subjects at all 147 k nucleotide positions and compared the different error suppression methods, including background polishing from iDES and the TNER method (Fig. 1). For TNER, we used α = 0.01, although the results were similar for α = 0.05. We also calculated the panel-wide error rate, which is defined as the number of nonreference allele reads (frequency < 5%, to exclude SNPs) divided by the total reads. The TNER method has the highest number of error-free positions and the lowest panel-wide error rate, demonstrating its superior specificity in reducing false positive error.

To test the sensitivity of the method, we used data from three healthy subjects who were not part of the background cohort. One subject had 10 unique private SNPs that were not shared by any of the healthy subjects. We performed an in silico experiment to dilute this subject’s data with those of the other two healthy subjects in a 1:250:250 ratio and assumed heterozygosity, producing an expected allele frequency of 0.1% for the 10 private SNPs. We found that both iDES and TNER (α = 0.01) were able to detect all 10 SNPs in this experiment.

### Model performance on simulated data

**Fig. 2:**
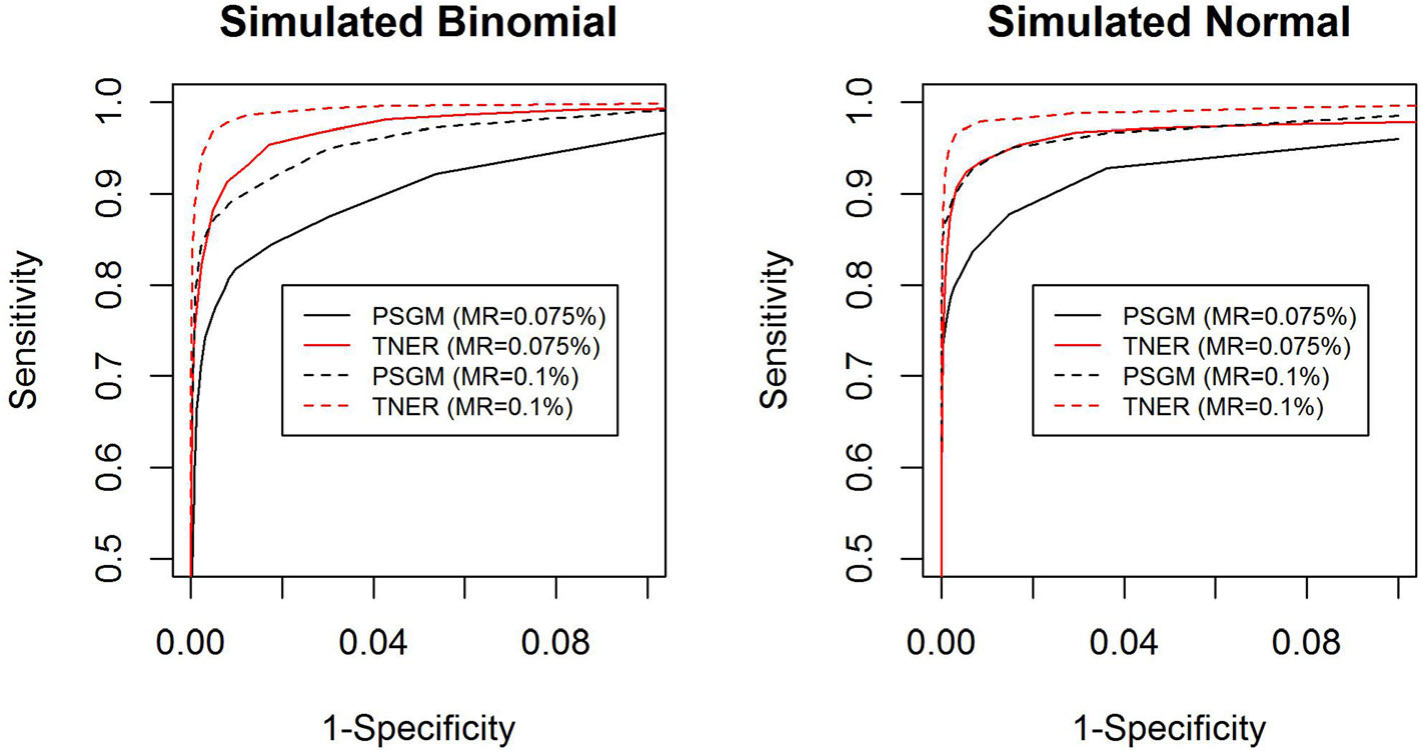
ROC curves for position-specific Gaussian model (PSGM) (black) and TNER (red) methods in simulated cfDNA data. Two mutation rates (MRs) were simulated: 0.075% (solid line) and 0.1% (dashed line), with a total coverage of 10000x at each position

To compare the performance of the position-specific background polishing method and the TNER method more rigorously, we evaluated their sensitivity and specificity at various detection thresholds using simulation studies (see the schematic in Additional file 1). The simulation used the average position-specific mutation error rate from the 14 healthy subjects as the BMER, which is a matrix of 147 k rows and four columns. Each column is a nucleotide that the reference base can mutate to, including the reference nucleotide, which is zero. We randomly selected 1,000 bases (rows) out of the 147 k total, and at each of the selected bases, a simulated allele frequency (simulated signal) was added to the existing BMER of a selected nonreference nucleotide (column). Specifically, for each of the 1,000 positions, there are three possible nonreference nucleotides to which it can mutate. We chose the nucleotide with the largest BMER value as the selected nucleotide to add the simulated signal. If the BMER had all zeros at this position, we used the first nonreference letter (A-C-T-G) as the selected nucleotide to add the signal. This updated BMER matrix is the same as the original matrix except that 1,000 rows have a signal added to a selected column. With the updated BMER matrix, we simulated the read counts with a total coverage of 10,000 per position using a binomial and a normal distribution. For the normal distribution, we simulated the allele fractions with the updated BMER as the mean and the square root of the BMER divided by 100 as the standard deviation. The read counts are calculated by multiplying the simulated allele fractions by the total coverage of 10,000 (round to whole number). The simulated counts were further split into forward and reverse strands with a random forward to reverse strand ratio centered at approximately 1. The TNER method and the position-specific Gaussian models from the iDES were then separately applied to the simulated data. As the true positives and true negatives were known, the sensitivity and specificity were calculated under various detection thresholds (α values). The receiver operating characteristic (ROC) curves in Fig. 2 compare the two methods in different scenarios. The TNER method performed better than the position-specific Gaussian model in all cases of data simulated under different distributions and different mutation rates (MRs), as shown by the ROC curves. Simulated mutation signals of 0.075 and 0.1% were chosen because they are close to the limit of detection for the methods when per base coverage is approximately 10,000x. Signals lower than the detection limit will be difficult to detect by either method.

**Fig. 3:**
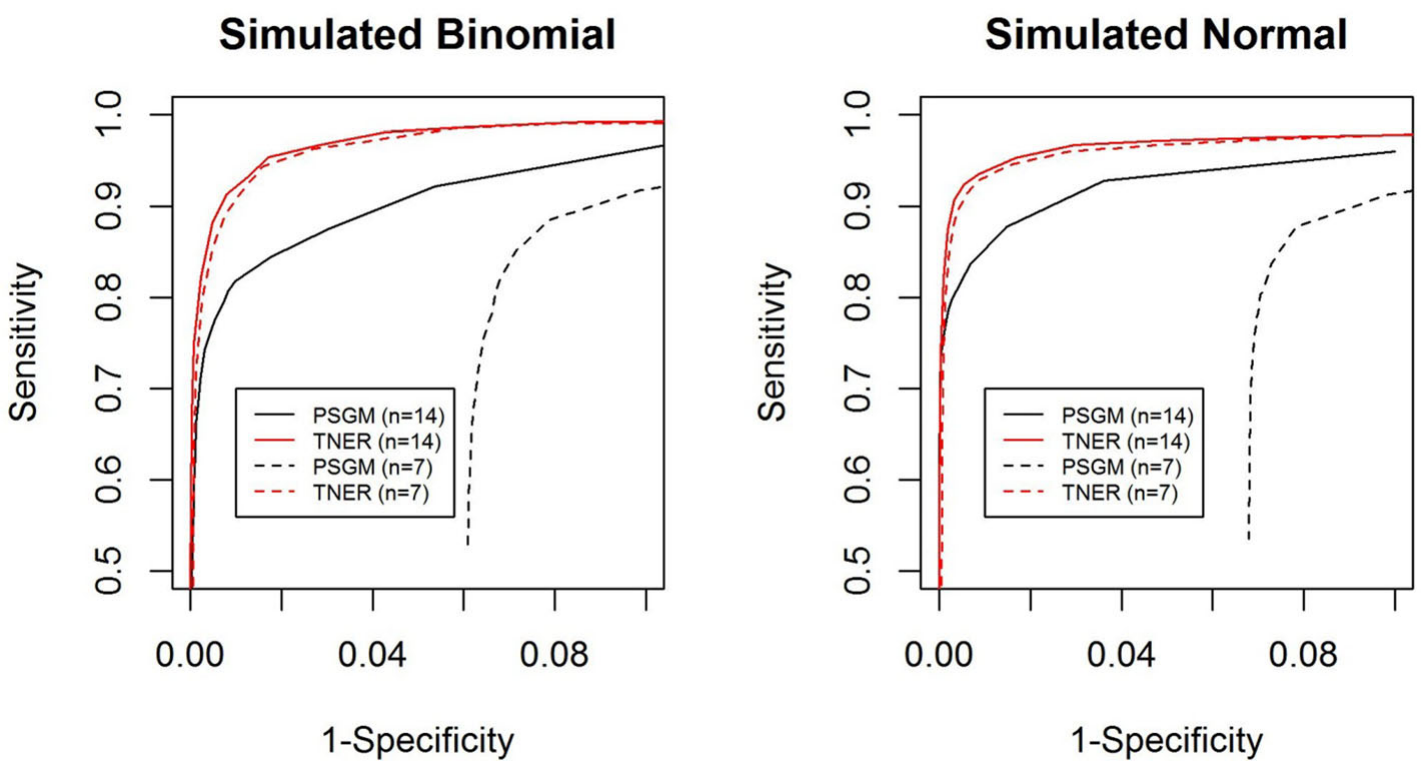
ROC curves of the position-specific Gaussian model (PSGM) (black) and the TNER (red) methods with different input numbers of healthy subjects: *n* = 7 (dashed line) and *n* = 14 (solid line). The mutation rate was 0.075%

One of the advantages of the TNER method is that it uses information from other positions with the same TNC through a Bayesian consideration and stabilizes the estimates of the BMER. Therefore, we would expect TNER to perform better than position-specific error models when the available sample size for healthy subjects is small. To evaluate the effect of healthy subject sample size on the performance of the mutation detection methods, we used half the available healthy subjects (*n* = 7) as our background mutation estimate and compared the results from both position-specific Gaussian models and TNER in the simulation studies. As expected, we found that a smaller sample size of healthy subjects did not substantially reduce the performance of TNER but greatly reduced the performance of the position-specific Gaussian method (Fig. 3) compared to other methods. This result clearly illustrates the robustness of the TNER method when the number of healthy subjects is small. In fact, we found that TNER can work even with 1–3 healthy subjects without excessively sacrificing performance.

## Discussion

In this study, we proposed TNER, a novel background polishing method for removing sequencing artifacts in panel sequencing data for liquid biopsy samples. The TNER method estimates background mutation errors from healthy subjects using a beta-binomial model to hierarchically incorporate both the tri-nucleotide-level error rate and the position-specific error rate. The additional information from the tri-nucleotide-level data helps stabilize the estimate of background errors and makes TNER more robust than the Gaussian-based, position-specific model used in iDES [17], especially when the number of healthy subjects is small. The results on both simulated and real healthy subject data demonstrated better performance of TNER than iDES in error reduction, indicated by substantially more error-free positions and a lower panel-wide error rate. TNER’s superior specificity in reducing false positive error can greatly benefit the downstreaming variant calling by general variant callers such as VarScan [30] or MuTect [31].

We could have used a dinucleotide context or a more complicated local sequence context, such as a pentanucleotide (2 flanking nucleotides on each side) or heptanucleotide (3 flanking nucleotides on each side) context. The larger local sequence context may provide a better model fit to the mutation error rate [32], but the increasing model complexity with the use of pentanucleotides (1,536 unique contexts) and heptanucleotides (24,576 unique contexts) becomes impractical for a targeted panel, such as the one tested here with a total of 147 k bases. The Bayesian prior parameter will not be well estimated due to the small number of bases within each context. The TNC provided a better fit than a dinucleotide context [33] but was less complicated than the larger local sequence context [32], thus providing a more balanced approach for a common NGS targeted panel.

**Fig. 4:**
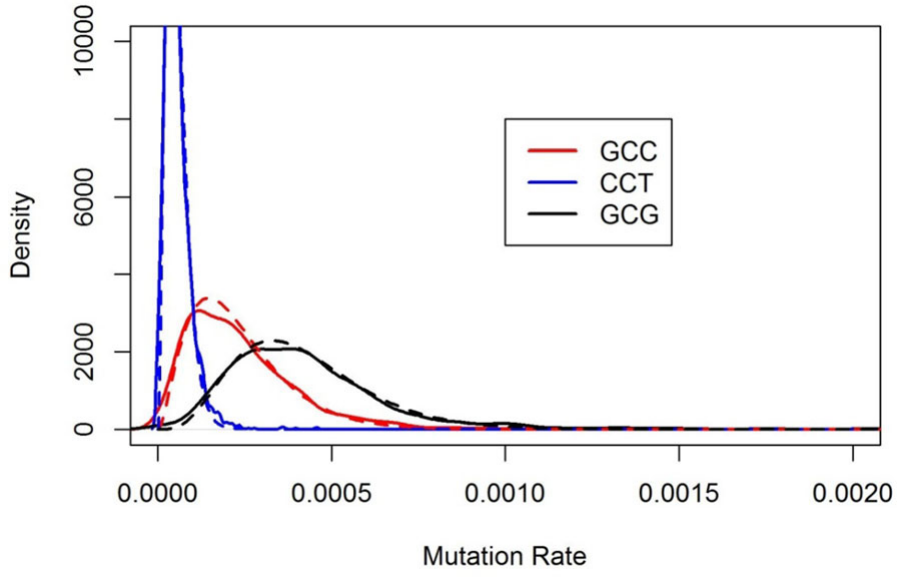
Examples of mutation error rate distribution of TNC with C-T substitution. Solid lines are the probability density of the average position-specific error rate within a TNC. The dashed lines correspond to the fit of a beta distribution using parameters estimated from the data

One of the assumptions in analyzing NGS data by TNER is that individual nucleotides within a TNC share a more similar mutation error rate than those between TNCs. We looked at the average mutation error rate from healthy subjects at the TNC level and compared the intra-TNC variability and the inter-TNC variability. Approximately 94% of TNCs have intra-TNC variability smaller than the inter-TNC variability. Figure 4 displays an example of three TNCs, all with C to T substitution, showing very different distributions. The dashed lines are the fit of beta distributions using the parameter estimates calculated by the method of moments. In general, the beta distribution fits the intra-TNC error rate very well.

In genomic data analysis, when the sample size is small, it is common to analyze data for individual genes using information from other genes. This approach is implemented in the *limma* method [34] for microarray data analysis and the DESeq method [35] for RNAseq data analysis. In our approach, we take advantage of the large number of bases shared in the same nucleotide context and use these data to model the individual base mutation error rate. We found that the TNER method improves the imprecise background estimate associated with small sample size at the individual base level.

Sequence data are read counts that are best described by distributions from discrete data families, such as the Poisson distribution or binomial distribution, particularly when the read count is low and the mutation frequency is very low, such as in ctDNA data. We found that the Poisson distribution fit the count data well in general. A more sophisticated distribution that considers over-dispersion and the zero-inflated nature of ctDNA data may further improve the method. The TNER method is a general statistical framework for detecting background sequencing noise, and in theory, it can be applied to any high-throughput NGS platform. Given the notable differences observed between the error profiles of Illumina platforms [36], we recommend that users always regenerate their own error profile from normal samples.

## Conclusions

Currently, ctDNA is rapidly becoming established as an important tool to supplement conventional biopsies for the early detection and molecular characterization of cancer and the monitoring of tumor dynamics. The TNER method provides a novel approach to effectively reduce background noise in panel sequencing data for more accurate mutation detection in ctDNA.

## Additional file

Additional file 1: **Figure S1.** Simulation schematic. (PNG 88 kb)

## Abbreviations

BMER: Background mutation error rate
cfDNA: Cell-free DNA cfDNA
ctDNA: Circulating tumor DNA
ddPCR: Droplet-digital PCR
NGS: Next-generation sequencing
SNV: Single-nucleotide variant
TNC: Tri-nucleotide context
TNER: Tri-nucleotide error reducer
