# Supplementary figures and images for: TNER: a novel background error suppression method for mutation detection in circulating tumor DNA
Source: BMC Bioinformatics. 2018 Oct 20;19:387. doi: 10.1186/s12859-018-2428-3 (PMC6195972; doi:10.1186/s12859-018-2428-3)

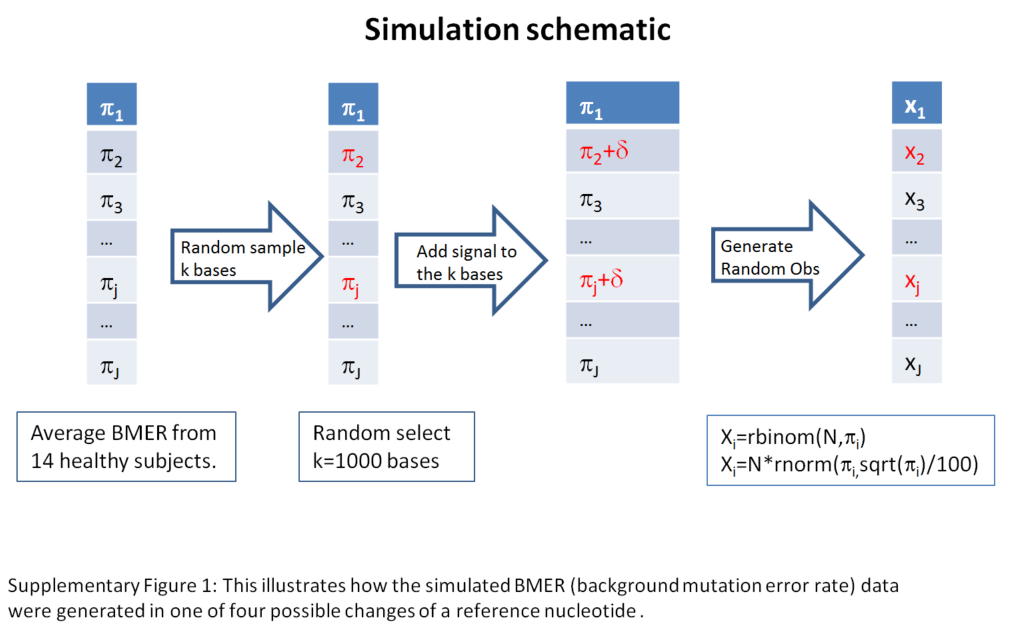

Supplement: Supplementary file 1 — Figure S1. Simulation schematic. (PNG 88 kb) [file 12859_2018_2428_MOESM1_ESM.png]
